# From barriers to opportunities from COVID-19 pandemic: Stakeholder perspectives on cervical cancer screening programs in LMICs of the Asia-Pacific region

**DOI:** 10.1371/journal.pgph.0003768

**Published:** 2024-10-04

**Authors:** Jieying Lee, Ida Ismail-Pratt, Dorothy A. Machalek, Suresh Kumarasamy, Suzanne M. Garland

**Affiliations:** 1 Asia Pacific HPV Coalition; 2 The Society for Colposcopy & Cervical Pathology of Singapore, Singapore, Singapore; 3 The Kirby Institute, University of New South Wales, Kensington, New South Wales, Australia; 4 Gleneagles Hospital Penang, George Town, Pulau Pinang, Malaysia; 5 Centre for Women’s Infectious Diseases, The Royal Women’s Hospital, Parkville, Victoria, Australia; 6 Department of Obstetrics and Gynaecology, University of Melbourne, Parkville, Victoria, Australia; The Chinese University of Hong Kong Faculty of Medicine, HONG KONG

## Abstract

Cervical cancer is preventable, yet it remains the fourth most common cancer in women globally. The highest incidence and mortality occur in low- and middle-income countries (LMICs), where over 70% of women have never been screened, and 58% of the cases are in Asia. While the COVID-19 pandemic caused significant disruptions to cervical screening programs, particularly for LMICs, there were opportunities that emerged from the pandemic that were enablers of program recovery. Stakeholders played key roles in materialising strategy into implementation. Therefore, in this study, we examined the barriers and facilitators to implementing recovery strategies from the stakeholders’ perspectives. We interviewed fifteen stakeholders from nine LMICs in the Asia-Pacific region directly involved in the implementation of the cervical screening program. A total of 23 barriers and 21 facilitators were identified, of which seven barriers and nine facilitators related directly to the pandemic. Pandemic-related barriers included movement restrictions, resource diversion, cancelled campaigns and training, deprioritisation of HPV prevention efforts, and a reduced health workforce. Stakeholders concurred that most barriers had predated the pandemic and remained as the pandemic eased. Conversely, the pandemic introduced facilitators such as means for targeted campaigns, improved understanding of viruses, accessible training with online platforms, better PCR testing capabilities, a shift in the government’s position towards preventive health services, and openness to HPV testing and self-swabs. The emerging facilitators offered opportunities to address some of the persistent barriers, such as limited cervical cancer awareness and insufficient healthcare providers in screening programs. However, effective implementation of these emerging facilitators requires improved communication and collaboration between policymakers and implementers to accelerate the recovery of screening programs in LMICs. Further work is necessary to align emerging facilitators with the health system goals and resource settings of each country in turning these opportunities into actions.

## Introduction

Cervical cancer is the fourth most common cancer in women globally [1]. Among the 604 000 new cases and 342 000 deaths recorded in 2020, the majority of patients (84%) and deaths (87%) occurred in low- and middle-income countries (LMICs), and 58% of the new cases occurred in Asia [2]. The disproportionately high incidence and mortality rates in LMICs are attributed to the persistent challenges of implementing and sustaining organised Pap-based cervical screening programs [3–5]. Therefore, cervical cancer is often not identified until it is well advanced. Moreover, access to effective treatments such as surgery, radiotherapy and chemotherapy is often limited or non-existent [3]. The high burden is further exacerbated by the high prevalence of HIV co-infection in some LMICs, which can directly impact the immune control of human papillomavirus (HPV) women [6]. For screening, it was estimated that 67% of women aged 20–70 years globally and 70% of women in LMICs had never been screened for cervical cancer [2]. The burden of cervical cancer mortality is expected to rise dramatically, and WHO predicts a 27% rise in mortality in LMICs compared to just a 1% increase in HICs by 2030 [7].

Cervical cancer can be effectively prevented with highly effective prophylactic vaccines delivered to pre-adolescent populations. The new cervical screening technologies based on the detection of oncogenic HPV by nucleic acid-based assays are more sensitive and objective in picking up preneoplastic lesions for appropriate treatment to avoid cancers [5, 8]. It enables the screening tests to be self-collected, which reduces the dependency on healthcare professionals and simplifies the scaling-up of screening programs. In view of these advances, WHO in 2018 has called for action globally to eliminate cervical cancer as a public health problem by working towards the target of fewer than four cases per 100 000 women and implementing three strategic pillars by 2030, i.e. 90% of girls vaccinated by the age of 15 years, 70% of women screened with high precision tests at least twice in their lifetime, and 90% of women with preneoplastic lesions or cancer be treated [5, 8].

The COVID-19 pandemic has exacerbated the existing challenges to reach the elimination targets as countries have diverted medical resources, professionals and equipment to manage the pandemic since Mar 2020 [4, 9]. School closures worldwide disrupted the delivery of HPV vaccines in those using school delivery programs and slowed down widespread rollout in many countries, particularly in LMICs [10]. The average HPV vaccination coverage dropped 25% across six countries that had introduced vaccines with Gavi support [11], and global HPV vaccine coverage decreased for the first time in 2020 [12]. For screening tests, there was an estimated decrease of 52% globally during the pandemic [13], and the reduction ranged from 14% in Bangladesh to 73% in Argentina [14]. These three strategic pillars of cervical cancer elimination have been extensively disrupted globally; the cancer screening rate declined, vaccination programs paused, and treatments were delayed.

**Fig 1:**
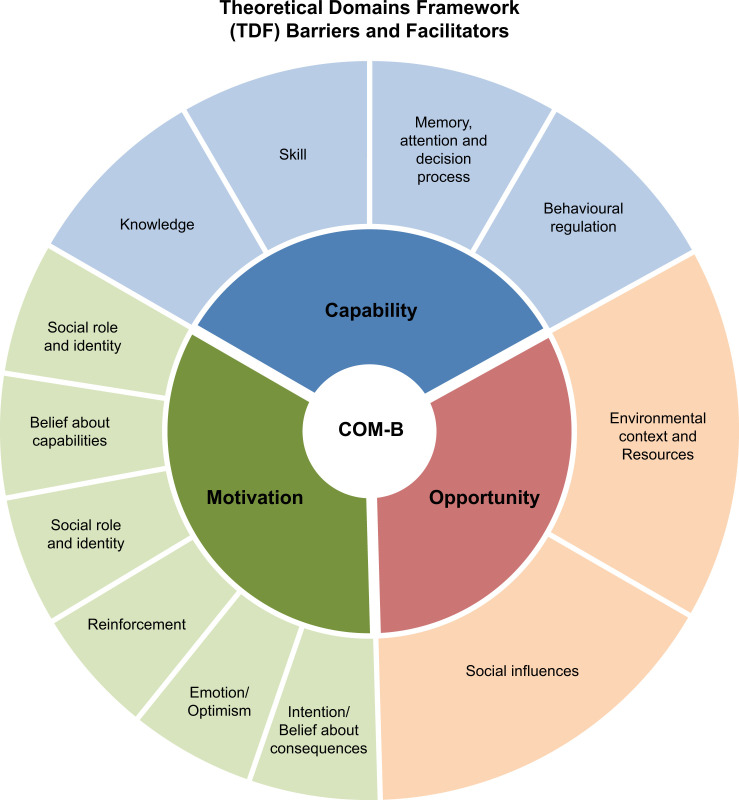
List of domains from Theoretical Domains Framework (TDF) and their categories in COM-B (Capability, Opportunity, Motivation and Behaviour) model.

To achieve the WHO target by 2030, there is an urgent need to coordinate recovery strategies in LMICs. A recent review identified 18 recommended recovery strategies, of which only six were implemented [9]. This review highlights the central role of stakeholders in materialising plans into implementation. The stakeholders, who worked closely with governments or the international communities, initiated the recovery strategies successfully during the pandemic. Therefore, in this study, we examined the barriers and facilitators to implementing recovery strategies from the stakeholders’ perspectives. All barriers and facilitators were analysed using the Theoretical Domains Framework (TDF) and discussed using the COM-B (Capability, Opportunity, Motivation and Behaviour) model [15, 16], see Fig 1.

## Methods

The stakeholders were recruited purposively to provide diversity of perspectives [17]. Invitations were sent to the authors’ contacts, through snowball recruitment strategy and corresponding authors’ contacts in the literature. The study was conducted between 19 August 2022 and 19 March 2023.

### Ethics statement

Written informed consent was obtained from all participants before the start of interviews, and all procedures followed were in accordance with the University of Melbourne ethics guidelines. The Human Research Ethics Low and Negligible Risk approval to conduct the study was obtained (Review reference 2022-24125-29964-4).

The categories of stakeholders were adapted from Kammi Schmeer’s Guidelines for Conducting a Stakeholders Analysis, created by Partnerships for Health Reform [18]. The stakeholders were divided into two groups based on two key characteristics, power, and leadership in cervical elimination efforts at the national, district or commune level: Decision-maker and Implementer.

- Decision-makers were key representatives of agencies who had the ability to impact cervical screening programs directly and held positions in the government or international organisations such as World Health Organization(WHO).
- Implementers were key representatives from Non-Governmental Organisations (NGOs), district or commune-level health management and did not hold positions in government or ministry. They were crucial in planning and executing programs to eliminate cervical cancer.

### Interviews

A semi-structured interview guide was developed to scope perspectives on key barriers and facilitators when planning and recovering cervical screening efforts following COVID-19 disruptions. The topic was developed using a recent systematic review of recovery strategies, see appendix for details. When appropriate, the interviewer would invite stakeholders to recommend any changes they would like to make to their existing cervical screening programs with rationales. The interviews were conducted through the online video conference platform, Zoom using a password-protected access link. The interviews were audio recorded and transcribed verbatim. The recordings were rendered anonymous during the transcription process to protect the anonymity of the stakeholders’ identities. Transcriptions were kept on a secure cloud server, and access was strictly controlled and only accessible to authors participating in the analysis.

### Coding and analysis

NVivo 11 (QSR International, Australia) was used to transcribe the audio record into text, and the identified text fragments in the transcripts were categorised into the 14 TDF domains and COM-B model.

Both deductive and inductive approaches to thematic analysis were used, as described by Atkins et al [16]. All the transcripts were read at least three times to ensure all the essential text fragments were included and allocated correctly. Regular discussions were held with the study team to resolve disagreements and discuss the themes that surfaced from interviews. The analysis process continued until the team felt they reached sufficiency in the data interpretation until they agreed on the interpretation of the data.

## Results

We approached 43 stakeholders, and 19 consented to interviews. Four could not attend due to scheduling conflicts, and 15 stakeholders (35%) participated in our study. We achieved a good mix of decision-makers (n = 7, 47%) and implementers (n = 8, 53%), as summarised in Table 1. The interviews lasted 46 min (+/- 10) on average. These stakeholders represented perspectives from nine countries; Bangladesh, Fiji, Indonesia, India, Mongolia, Nepal, Pakistan, Sri Lanka and Vietnam. In all these countries, cervical screening was opportunistic, meaning women or healthcare professionals could initiate participation without any organised invitation system in place. Sri Lanka and Mongolia implemented a selective invitation strategy for women aged 35 and 45 to participate in screenings. Bangladesh established screening registries to monitor uptake and implemented a selective outreach strategy in towns with lower screening rates.

**Table 1:** Characteristics of stakeholders interviewed.

		Decision-makers	Implementers
**Gender**	Female	5	8
	Male	2	-
**No. of years in role(s)**	< 10 years	3	2
	10–20 years	1	3
	> 20 years	3	3
**Current role(s) related to cervical cancer elimination efforts**	Holding key leading positions at NGOs, agencies or professional associations	5	8
	Advisors to the Ministry or holding government positions	7	-
	Holding decision-making positions at international organisations such as WHO	2	-

We identified 23 barriers to implementing cervical screening programs, Table 2. Sixteen of these barriers existed before the pandemic, while seven emerged due to movement restrictions, limited resources, and fewer opportunities during the pandemic. The barriers that existed before the pandemic were related to five domains: *1 Knowledge*, *2 Skill*, *3 Memory*, *attention and decision process*, *4 Social influence*, *and 5 Environmental context and resources*. The other seven barriers were associated with four domains, 1 *Environmental context and 2 Resources*, *3 Knowledge*, and *4 Memory*, *attention and decision process*.

**Table 2:** List of barriers and facilitators grouped by Theoretical Domains Framework (TDF) and their categories in COM-B (Capability, Opportunity, Motivation and Behaviour) model.

		23 Barriers		21 Facilitators	
COM-B	TDF Domains	16 Persistent	7 Emerging	12 Persistent	9 Emerging
**Capability**	Knowledge	2	1	1	3
	Skill	5	-	-	2
	Memory, attention and decision process	2	1	-	-
	Behavioural regulation	-	-	1	-
**Opportunity**	Environmental context and Resources	5	5	2	-
	Social influences	2		-	2
**Motivation**	Intention/ Belief about consequences	-	-	-	1
	Emotion/ Optimism	-	-	2	1
	Reinforcement	-	-	2	-
	Goals	-	-	1	-
	Belief about capabilities	-	-	1	-
	Social role and identity	-	-	2	-

There were 21 facilitators supporting the cervical screening programs, Table 2. Twelve of these facilitators were in place before the pandemic, while nine emerged due to innovations introduced during pandemic. The facilitators in place before the pandemic were related to eight domains– 1 *Knowledge*, *2 Social influence*, *3 Social role and identity*, *5 Reinforcement*, *6 Emotion*, *7 Optimism*, *and 8 Behavioural regulation*. The other nine facilitators were related to six domains—1 *Knowledge*, *2 Skills*, *3 Intentions*, *4 Optimism*, *5 Belief about consequences and 6 Social influences*.

In the following subsections, we elaborated on the barriers and facilitators identified by stakeholders. Each subsection includes a table of barriers and facilitators mapping to the Theoretical Domains Framework, accompanied by at least one sample quote (Q) from a decision-maker or implementer.

### Barriers that emerged as results of COVID-19 measures

During the pandemic, there were three barriers related to *Environmental context and Resources*, Table 3. All stakeholders mentioned that movement restrictions posed challenges for women accessing healthcare facilities (see Q1). Most stakeholders noted that the diversion of resources towards COVID-19 management affected the availability and allocation of healthcare professionals, service providers, and resources for cervical screening (see Q2). Consequently, cervical screening programs were either cancelled or delayed (see Q3) which was related to *Knowledge*. Some implementers stated that training and refresher courses were cancelled as healthcare professionals and service providers were busy managing COVID-19, further worsening the need for skilled healthcare professionals in screening programs (see Q6). One of the implementers explained that despite requests for resources to resume HPV-related prevention efforts, priority was given to other concerns, such as COVID-19 and children’s vaccination programs (see Q7) which was related to *Memory*, *attention and decision process*.

**Table 3:** Barriers that emerged as a result of COVID-19 measures.

TDF Domains	Barriers	Sample Quotes	Decision-makers	Implementers
Environmental context and Resources	Movement restriction during the pandemic	“there was lockdown so people could not also access the health facilities due to a lack of transportation” [Q1]	✓	✓
	Diverted resources	“Human resources are diverted towards the COVID-19 management and even the service provider who is providing the services for cervical screening” [Q2]	✓	✓
	Campaign was cancelled, and screening was delayed	“all regular screening programs were just postponed” [Q3]	✓	✓
	Even fewer healthcare professionals as COVID-19 eases	“mass exodus from healthcare professionals after COVID” [Q4]	✓	
	Even limited resources as COVID-19 eases	“government is struggling for the resources and also manage the human resource issues” [Q5]	✓	
Knowledge	Training and refresher courses cancelled	“They were so busy that almost all the medical training programs were cancelled. Completely cancelled!” [Q6]		✓
Memory, attention and decision process	No priority for HPV-related prevention efforts	“They say, Oh! We have many other priorities like COVID and children’s vaccination programs.” [Q7]		✓

As movement restrictions were lifted in most countries, a few decision-makers identified two additional barriers related to *Environmental context and Resources*. A decision-maker witnessed a mass exodus of professionals from public to private healthcare institutions, leading to even limited human resources available to support existing cervical screening programs (see Q4). Other decision-makers also identified similar constraints in human resources within their governments due to economic struggles during COVID-19 (see Q5).

### Facilitators that emerged due to the COVID-19 pandemic

Although the pandemic brought challenges, it also introduced facilitators to screening programs, as evidenced by the quotes by decision-makers and implementers in Table 4. Due to the movement restrictions, online platforms such as webinars and social media became alternative and reliable venues to raise awareness and educate the public. One implementer highlighted the efficacy of online radio talks, which garnered over 10,000 subscribers during the pandemic (see Q8), while other implementers mentioned using webinars to educate the public. Decision-makers shared that social media campaigns were good for disseminating knowledge to targeted audiences. One decision-maker had success with social media campaigns specifically aimed at individuals who are thirty-five and forty-five years old (see Q9), and another decision-maker targeted specific regions by employing campaigns in local languages, thereby encouraging attendance among the targeted audience. Some stakeholders pointed out that the unique circumstances of the pandemic enabled more online training, as healthcare professionals were confined at home (see Q11). Online training has become a widespread practice, revolutionising training after the pandemic. This shift allowed participants to engage from anywhere, at their convenience, breaking down the barriers of physical distance.

**Table 4:** Facilitators that emerged due to the COVID-19 pandemic.

TDF Domains	Facilitators	Sample Quotes	Decision-makers	Implementers
Knowledge	More channels to reach the public	“We have a series of radio talks, and twice a month, volunteers take on topics of interest and speak about them…I think in the last two years of COVID, particularly, we have had over 10,000 users.” [Q8]	✓	✓
	More targeted campaigns	“We have a social media campaign. And we send messages to those who are thirty-five and forty-five years” [Q9]	✓	
	A better understanding of the virus after COVID-19	“Because they understood that having an immunity, we don’t get COVID. It’s a virus. So, the virus became a name. It is a very common name in each and every household.” [Q10]	✓	✓
Skill	COVID-19 elevate preparedness with more online training	“COVID was the time when people were arrested in their house. There was a lot of time. . .reach out to each and every gynaecologist” [Q11]	✓	✓
	Better PCR capability after COVID-19	“all provinces, all district now have the possibility to have molecular biology testing” [Q12]	✓	✓
Intention/ Belief about consequences	Shift in government position towards preventive health services	“when the COVID situation is getting a bit better, the government is starting to think about how to improve this other health services, including screening programs.” [Q13]	✓	
Optimism	Right time to start HPV testing	“it’s high time that we try to interchange HPV” [Q14]		✓
Social influences	More open to prevention after COVID-19	“change occurring in the mentality of the patients. …but the mentality of the public, and even the public figures, policymakers, and stakeholders. So, a lot of attention started pouring in for prevention.” [Q15]	✓	✓
	More open to self-swabs	“Now many people have learned to do the nasal swabs, so in a similar way, they could take a vaginal swab and test themselves whether they are HPV positive or negative.” [Q16]	✓	✓

Most stakeholders observed that the pandemic improved public awareness and understanding of the virus, diagnostics, vaccines and immunity (see Q10). They explained that the public recognised the link between immunity and the virus, for example, COVID-19 and vaccines and how HPV vaccination can prevent certain types of HPV infections that could lead to cervical cancer. This enhanced awareness and knowledge helped the stakeholders to better explain the importance of HPV vaccination and HPV screening tests to their patients, public and policy makers. Hence, this has potentially influence positive public attitudes towards prevention efforts related to cervical cancer (see Q15). Several stakeholders also noted increased public openness to self-swabbing for screening purposes, which spurred optimism among some implementers, suggesting it is the opportune moment to introduce HPV testing or self-sampling tests (see Q14 and Q16). Some decision-makers also indicated a shift in government focus towards preventive health after the pandemic and saw opportunities to propose plans related to cervical cancer elimination (see Q13).

In other respects, most stakeholders affirmed that countries now have an enhanced capacity for molecular biology testing by introducing Polymerase Chain Reaction (PCR) machines and resources for detecting COVID-19, which could be pivoted for HPV testing (see Q12).

### Persistent barriers that did not change during and after COVID-19 pandemic

All stakeholders shared that most barriers to cervical screening programs persisted before the pandemic and remained as the pandemic eased, Table 5. They agreed that the key barrier was limited understanding and awareness of cervical cancer (see Q17). A few implementers pointed out that the disease was not as visible as breast cancer, making it more difficult for the general population to grasp the impact of cervical cancer (see Q18). Even when well-known celebrities succumbed to cervical cancer, women often struggled to perceive the disease as a personal risk due to the inability to observe the affected organ visually. One implementer mentioned that some women remained unaware of cervical cancer until they were diagnosed. Many stakeholders shared that more attention could be used on cervical cancer awareness (see Q19). However, most stakeholders noted that cervical cancer was not high on the list of concerns for gynaecologists, who typically devoted more attention to obstetrics (see Q20). They also conveyed difficulties in motivating clinicians, as individual priorities and workloads often take precedence.

**Table 5:** Persistent barriers that did not change during and after COVID-19.

TDF Domains	Facilitators and barriers	Sample Quotes	Decision-makers	Implementers
Knowledge	Lack of awareness among the public	“general population has only limited understanding and appreciation of the importance of cervical screening” [Q17]	✓	✓
	Difficult to understand the extent of cancer	“they cannot see the disease like breast cancer…what happened in the area(cervical) can only be seen by the doctor” [Q18]	✓	✓
Memory, attention and decision process	Not enough attention on cervical cancers	“Lack of awareness is a chronic problem. That’s why it’s not similar to COVID-19 awareness, it could not attract the attention yet.” [Q19]	✓	✓
	Lack of emphasis on the prevention of cervical cancer	“A lot of women, especially in government, hospitals, in the OBGYN department, the major burden would be doing the obstetrics that is delivering the babies. Less importance is given to the preventive part.” [Q20]	✓	✓
Skill	Insufficient trained healthcare professionals	“not only cytologists but many healthcare professionals, really big turnover” [Q21]	✓	✓
	Overstretched healthcare professionals	“They are very overburdened and are taking the responsibilities of many things. We try to focus on what we can, and they are not only focusing on the Cervical cancers, so that this is also another challenge” [Q22]	✓	✓
	Challenging to train and ensure quality in VIA	“VIA is really, really very hard for us to do the training and the training sometime is not in good quality.” [Q23]	✓	✓
	Limited lab support for cytology	“not every district has the pathologist to the evaluation” [Q24]		✓
	Limited lab support for HPV testing	“major labs which we use, both said that We are not in a position to report the self-swabs” [Q25]		✓
Social influence	Stigma associated with seeking medical care	“The concept of prevention does not exist in our country.” [Q26]	✓	✓
	Stigma related to the screening method	“We feel that we are alright, why should we have this shameless examination?” [Q27]	✓	✓
Environmental context and resources	Lack of information system for managing and tracking women	“We don’t have a comprehensive system of managing and following up who had Cervical screening the last year, so that is the biggest challenging thing” [Q28] “Even though there are guidelines, there are people who say that they’re doing it, but I don’t think much is happening at the ground level” [Q29]	✓	✓
	Expensive reagents	“HPV is better, but we don’t have money for that.” [Q30]	✓	✓
	Need resources and training to change from VIA to HPV	“needs to spend more money on all these job creation, and maybe more (training) camps, more activities” [Q31] “We cannot leave the VIA suddenly.” [Q32]	✓	✓

The other barrier was the shortage of trained healthcare professionals to support screening programs, as resources were constrained in LMICs. Some decision-makers observed high turnover rates among doctors, nurses, and cytologists (see Q21). In addition, these healthcare professionals had to manage many programs besides cervical screening (see Q22).

Some stakeholders recognised the difficulty in setting up quality checks for screening programs, especially for subjective screening methods (see Q23). This strain was particularly notable when screening with Visual Inspection with Acetic Acid (VIA) in resource-limited areas where only a trained professional might be on site, with no other trained staff to provide a second opinion. There were issues with other tests, such as cytology and HPV tests. Implementers highlighted the lack of quality control for cytology services in some laboratories and limited access to laboratory services in rural districts (see Q24), while others drew attention to the insufficient laboratory support for HPV testing, especially self-sampling tests (see Q25).

The social stigma was another barrier. Almost all stakeholders shared that the concept of public health screening was not widely practised, contributing to a lack of opportunities to screen and potential delays in seeking medical care (see Q26). An implementer shared that people do not see doctors until they feel sick. The screening methods for cervical cancer also made women defer screening, as the tests involved exposing private areas to healthcare professionals and the discomfort associated with inserting the speculum or swab (see Q27).

Within the environmental context and resources domain, some stakeholders acknowledged the critical yet absent information systems necessary to effectively monitor the screening programs’ impact and reach (see Q28). Implementers further highlighted the importance of monitoring by highlighting the discrepancies between action plans and the actual implementations (see Q29).

In terms of implementing HPV testing, all decision-makers and implementers acknowledged its superiority and advantages in diagnosis, while expressing concerns about the cost of reagents (see Q30). They favoured the sensitivity of HPV testing compared to VIA and cytology and the feasibility of scaling up cervical screening programs with HPV tests. However, to transition from VIA or cytology to HPV testing, many stakeholders highlighted the need for a gradual shift or cost-effective analysis to justify the change (see Q32). In addition, they noted that extra resources would be needed to train sufficient staff to support the transition from VIA to HPV testing (see Q31).

### Persistent facilitators that did not change during and after the COVID-19 pandemic

Despite facing numerous obstacles to implementing screening programs, decision-makers and implementers successfully advanced their agenda and made progress in eliminating cervical cancer within their districts or countries. They listed 12 facilitators in Table 6. Effective communication emerged as the primary facilitator to overcome the lack of awareness (see Q33). All stakeholders emphasised the importance of advocacy materials and training for the public and healthcare professionals in raising awareness. They stressed the necessity of educating women and their families about the significance of cervical screening to encourage women not to skip their screening tests.

**Table 6:** Persistent facilitators that did not change during and after COVID-19.

TDF Domains	Facilitators and barriers	Sample Quotes	Decision-makers	Implementers
Knowledge	Good channels to raise awareness and education	“advocacy materials are really important and the educational resources are really important, both for healthcare professionals and for the woman.” [Q33]	✓	✓
Reinforcement	Integrating with other programs	“Basic health service package, which includes the Cervical screening service” [Q34] “We offer the entire gamut (from our screening buses). We do a chest X-ray, blood profile, and oral checkup; the men go to a male physician for a physical checkup. And the women go to a gynaecologist who does a breast examination and a pap smear.” [Q35] “Screen the mothers and vaccinate the daughters” [Q36]	✓	✓
	Recognition of best performers	“At the performance appraisal, we used to give them an award to the best-performing districts, to the best performing public midwife, best performing medical officers.” [Q37]	✓	
Goals	National guidelines to guide actions	“they have the national guidelines and action plan for us, and to do aside the duty for cities and provinces.” [Q38]	✓	✓
Behavioural regulation	External push from NGOs to set and track progress	“We need to provide some data to GAVI. So, they said, “Okay! We are going to start a project.” [Q39]	✓	
Belief about capabilities	Extensive network of community health workers with knowledge of cervical cancer and determination	“good network of public health volunteers at the commune level” [Q40] “They’re not doctors, or they’re not even health personnel, but they have got a good knowledge about cervical screening. So they motivate women to come for the screening camps.” [Q41] “Community health worker and nurses can move mountains, and I’ve watched them do it.” [Q42]	✓	✓
Social role and identity	Champion in preventive efforts	“I feel it’s part of my work.” [Q43]		✓
	Appointing advocates	“identified as ambassadors for preventive oncology and they become the reference for their vicinity doctors or areas around them” [Q44]		✓
Emotion/Optimism	Passionate stakeholders	“have your intention, and a deep passion burning into your tummy and your belly” [Q45]		✓
	Perseverance	“You keep persisting year after year after year, they get this letter in the month of November within the first five days of November. They know that you mean business.” [Q46] “You just keep doing it. Keep doing it. Keep doing it. And if water can wear stone down, and they are all humans.” [Q47]		✓

The other facilitator was the establishment of national guidelines. All stakeholders mentioned that the guidelines played an instrumental role in shaping action plans at both the national and local levels, including provinces or districts (see Q38). Some decision-makers suggested complementing national guidelines and action plans with information system screening indicators to ensure that these programs’ impact can be measured for accurate allocation of resources and planning. Decision-makers who successfully measured outcomes noted that non-governmental organisations were facilitating and prompting the government to establish these information systems for data related to screening and or vaccination programs (see S39).

Another facilitator was to integrate cervical screening with other programs such as the basic health service package (see Q34). The benefits of integration include increased motivation for women to go for screening and to share the limited resources for preventive healthcare. One implementer explained that women would be more inclined to participate in overall health screening programs instead of cervical screening programs focusing on individual body parts (see Q35). Another successful example from two implementers was the strategy to screen the mother and vaccinate the daughter simultaneously. During the school-based HPV vaccination program, the girls would get vaccinated with HPV, while their mothers get screened for cervical cancer (see Q36).

In seven of the nine countries interviewed, stakeholders consistently identified the role of community health workers as a key facilitator. Most stakeholders noted an extensive network of community health workers at the grassroots level assigned to each public health unit (see Q40). Although these workers might not have the same medical training as nurses or doctors, all stakeholders agreed they had sufficient healthcare knowledge and community understanding to educate and encourage women to participate in cervical screening programs (see Q41). They were described as motivated and acted as catalysts in promoting various screening programs (see S42). One implementer leveraged and supported the community health workers to visit households to educate women on cervical cancer and also collect HPV swab samples from women.

We also noticed many implementers were enthusiastic champions of cervical cancer elimination efforts, viewing these efforts as part of their professional identity (see Q43). They harboured a deep passion for advocacy that kept them relentless in the face of indifference among other healthcare professionals (see Q45). They continuously promote cervical screening through various means, such as sending yearly reminder emails to gynaecologists and running mobile clinics for health screening (see Q46). Almost all implementers firmly believe that passion is a key facilitator for advancing their screening campaigns and overcoming implementation barriers (see Q47). Some implementers even extended their passion to other clinicians by appointing them as ambassadors for cervical cancer elimination efforts (see Q44). As ambassadors, these clinicians served as references for other healthcare professionals in their communities, advocating for awareness and education within their locales. One decision-maker shared that they encouraged advocacy among healthcare providers by rewarding good performance and recognising efforts with awards (see Q37).

### Decision-makers and implementers have differing views on resources

Among the persistent barriers and facilitators, we observed notable differences in views between decision-makers and implementers regarding funding and resources in the Environmental context and resources domain, Table 7. Most decision-makers reported receiving government funding and expressed optimism when asked if their governments would support initiatives related to cervical screening programs (see Q48). In contrast, most implementers identified limited funding as a major barrier. Most implementers financed their cervical screening programs with donations from companies and NGOs (see Q49). Likewise, regarding resources, implementers reported a scarcity of equipment for treatment and referral centres for women requiring colposcopies (see Q52 and Q53). Conversely, decision-makers were more positive about the potential increase in resource allocation, especially in preventive health efforts (see Q50). They were also more aware of government plans to expand cervical screening programs (see Q51).

**Table 7:** Decision-makers and implementers have differing views on these barriers and facilitators.

TDF Domains	Facilitators and barriers	Sample Quotes	Decision-makers	Implementers
Environmental context and resources	Sufficient funding	“we got funding from the government. So I think government is now more trying to support these kinds of projects.” [Q48]	✓	
	Limited funding	“Money is again, a major block. Funding is the major issue. So this funding is the major issue, which I’m able to do up to some extent, with the help of the charities” [Q49]		✓
	Lack of equipment for treatment or referral	“referral link is what we are lacking” [Q52] “cryo surgery is also not easy, because we need the gas, we need the tools” [Q53]		✓
	Resources for screening and plan to have more logistic support	“redundancy for public health clinics in the country. And a lot of preventive or public health activities are going on. So one of them is Cervical screening” [Q50] “logistical supply to increase the screening and treat program” [Q51]	✓	

## Discussions

**Fig 2:**
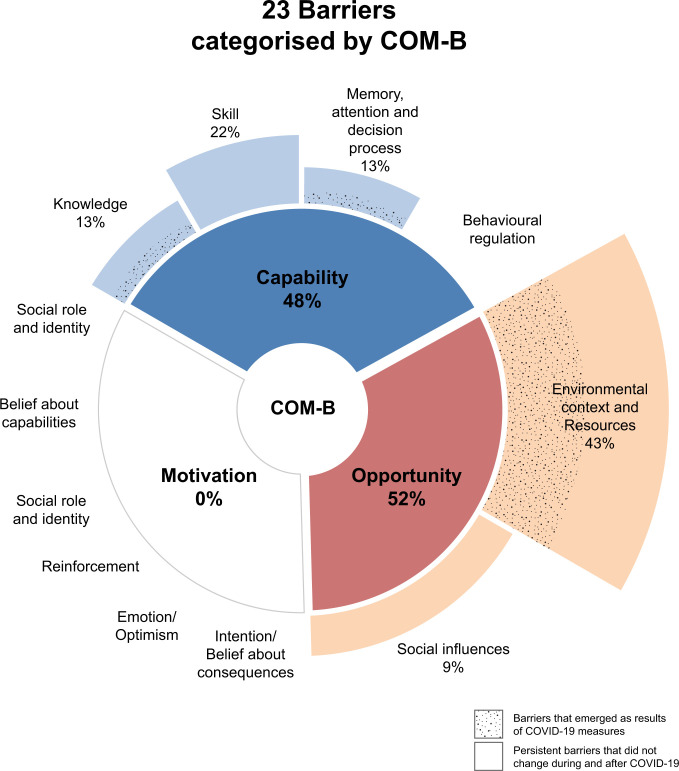
Barriers categorised by COM-B model.

**Fig 3:**
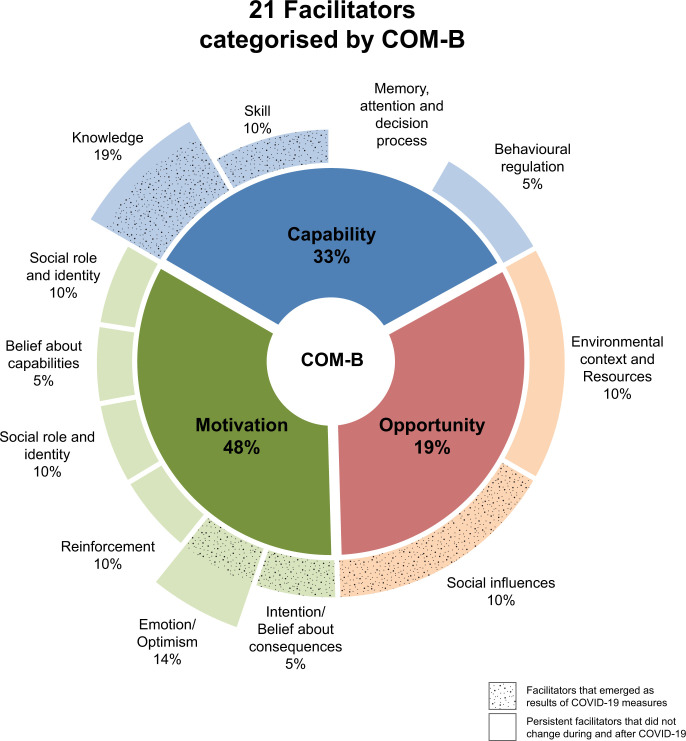
Facilitators categorised by COM-B model.

After interviewing 15 stakeholders, comprising seven decision-makers and eight implementers, we identified 23 barriers and 21 facilitators associated with implementing cervical screening programs. Of these, seven barriers and nine facilitators were related to COVID-19. In order to have a deeper understanding of these barriers and facilitators, we used the COM-B model to group the TDF domains into three categories: *Capability*, *Opportunity*, *and Motivation*, as summarised in Figs 2 and 3. All identified barriers were mapped to *Capability* and *Opportunity*, with none mapped to *Motivation*. The barriers that emerged as a result of COVID-19 measures predominantly fell under *Opportunity* with the remainder to *Capability*. Notably, half of the identified facilitators were mapped to *Motivation*, and the remainder to *Capability* and *Opportunity*. Most facilitators that emerged due to the pandemic were associated with *Capability and Opportunity*.

The Motivation of the COM-B model, defined as reflective and automatic processes that drive actions [15], emerged as the key category facilitating the cervical screening programs across all the countries we interviewed. The commitment of stakeholders involved in our study was evident. Their engagement in screening initiatives showcased their dedication to cervical cancer prevention before and after the pandemic. Although there may be a bias towards respondents who volunteered for our study, this level of motivation was uniformly observed across all nine countries included in our study.

In the subsequent section, we will explore alignments between facilitators and barriers identified by stakeholders within the *Capability and Opportunity* categories. The discussions were summarised in Table 8 to offer actionable recommendations for future interventions.

**Table 8:** Opportunities to accelerate cervical screening programs.

1. Targeted awareness and education campaigns via online social media platforms 2. Enhance cervical screening and treatment capabilities through accessible online or blended learning 3. Integrate cervical screening programs with related screening such breast cancer screening or HPV vaccination programs for extended reach and opportunities to share resources 4. Empower community health workers to expand the reach and impact of cervical screening programs 5. Conduct analysis studies to evaluate the costs and resources required to transition from existing screening methods to HPV-based testing as the primary test 6. Support countries in developing roadmaps for the introduction of HPV-based tests into care algorithms with a focus on timely follow-up processes and referrals to treatment centres 7. Leverage existing or COVID-19 information systems to monitor cervical screening programs and optimise the allocation of resources 8. Foster communication and collaboration between policymakers, decision-makers, and implementers to accelerate efforts towards cervical cancer elimination

### Capability

*Capability* of the COM-B model refers to the psychological and physical capacity needed to engage in an activity [15], such as having the necessary knowledge or skills for implementing cervical screening programs. The key persistent barriers that did not change during and after COVID-19 were *Skills and Knowledge*, see Fig 2. Stakeholders gave examples of key challenges such as insufficiently trained healthcare professionals who were already overstretched, difficulty in maintaining quality in VIA, limited laboratory support for cytology and HPV testing and a general lack of awareness and understanding of cervical cancer. Positively, the pandemic opened new opportunities for targeted public engagement through online platforms and enhanced the public’s knowledge of viruses, facilitating easier HPV and cervical cancer counselling. Also, the pandemic led to more accessible healthcare professionals training via online platforms and improved molecular testing capabilities in many countries, including the availability of PCR machines and laboratory capabilities.

#### Paradigm shift in raising awareness

The lack of awareness and understanding surrounding cervical cancer has been a persistent issue [19, 20].

Numerous studies in similar resource settings had identified the primary reason for women not undergoing cervical screening as a lack of awareness [21, 22]. These studies found that screening uptake increased after introducing interventions to improve knowledge of cervical cancer and its screening. Awareness and education campaigns were previously done via printed media or when women received services from healthcare institutes. With increased familiarity with using social media to receive health-related information during the pandemic, some implementers shifted their efforts to initiate more campaigns using online platforms. Examples included hosting radio talks, sharing videos on WhatsApp (an instant messaging mobile application) and conducting social media campaigns. Online platforms diversified the channels available to increase literacy on cervical cancer and reduce hesitancy related to screening tests. Additionally, social media platforms offered the advantage of tailoring campaigns to specific age groups targeted for screening, particularly those in the age ranges related to 35 and 45 years old. By focusing campaigns more precisely, it was possible to economise on limited budgets and extend reach to more of the intended participants.

#### Overcoming staffing challenges with accessible learning and program integration

The shortage of trained healthcare professionals was highlighted by decision-makers, implementers and also in literature [13, 23, 24]. During the pandemic, healthcare professionals scheduled for training workshops were diverted to manage the crisis, further worsening the shortage of trained personnel in screening programs. However, the pandemic showcased opportunities to make training more accessible with digital tools and new ways to train more healthcare professionals to support cervical screening programs. Some implementers seized the unique opportunity of movement restriction to increase training during the pandemic and disseminate knowledge related to cervical cancer prevention through webinars and websites. Similarly, a group in Mozambique introduced blended virtual learning training during the pandemic and succeeded [25], while other literature recommended introducing digital platforms and more online workshops for stakeholders, governments, academics and healthcare institutions to accelerate knowledge exchange [23, 26].

The other opportunity highlighted by implementers was the integration of the cervical screening programs with the school-based HPV vaccination programs, echoing recommendations from the literature to integrate with HIV clinic [27] or integrating HPV vaccination with other adolescent vaccination programs. However, literature and stakeholders stressed the importance of establishing effective communication between program implementers and decision-makers for successful integration. Decision-makers and implementers pointed out that healthcare professionals in LMICs were often overstretched, underscoring the need for conversations to set realistic objectives and consider allocating shared resources carefully. Furthermore, stakeholders noted that this strategy might not suit their context due to post-pandemic resource limitations and a reduced pool of trained professionals.

#### Empowering community health workers to extend cervical cancer education and screening

In seven of the nine countries we interviewed, large communities of health workers supported the cervical screening programs at the community level. Stakeholders in regions with a scarcity of trained healthcare professionals highlighted the critical role of community health workers in enhancing awareness and education about cervical cancer’s significance. One implementer was conducting a pilot study where community health workers collected HPV swab samples from women and provided education on cervical cancer, thereby broadening the screening program’s impacts. Similar initiatives leveraging community health workers to engage under-screened populations in hard-to-reach areas were observed in northern Bangladesh and El Salvador [10, 28–30]. The WHO also advocated for community health workers as a key strategy for reaching the most marginalised populations to achieve Universal Health Coverage and reduce health inequities [31]. Similarly, in a scoping review, community health workers participated in community education, outreach, and awareness activities in 14 out of 15 studies, while in four studies, they performed cervical screening tests and supported specialist healthcare professionals [32]. Thus, with additional training and support, community health workers would be the catalyst in expanding screening programs’ reach and impact.

#### Enhancing molecular testing capabilities for more sensitive and objective screening

The WHO recommends HPV testing over cytology or VIA for primary cervical screening for all settings [33]. HPV tests are more sensitive and objective screening tests [34, 35]. However, before the pandemic, there was a lack of molecular testing infrastructures in LMICs. The expansion of laboratory capabilities for COVID-19 testing had equipped countries with the necessary equipment for HPV testing, yet reagents remained challenging. Stakeholders reported that the cost of reagents was high. The pandemic showcased a global pooled procurement mechanism, where international organisations such as the COVID-19 Vaccines Global Access (COVAX) system pool buying power from participating economies and provide volume guarantees across a range of promising vaccine candidates to support participation from various countries. For the HPV test to be the primary screening test in more LMICs, there should be similar organisations to negotiate the prices of HPV tests to increase accessibility [36].

Stakeholders also pointed out that adopting HPV testing would necessitate an overhaul of the service delivery model and an update of screening policies and guidelines, necessitating additional resources such as training, more laboratory personnel, and support staff. Although cost-effectiveness studies conducted in LMICs had confirmed the efficacy of HPV tests [7, 37, 38], the challenge was to justify the additional expenditure to setup new testing method. Also, there were challenges to integrate HPV testing into the algorithms of care and how it would be followed up and referred to treatment centres, especially in resource-limited settings. Learning from the global coordinated response to COVID-19, a top-down approach to implementing the global strategy in LMICs would result in a lack of ownership and affect the success of delivery [39]. Hence, the integration of HPV testing into algorithms of care would need to be contextualised based on the different resource settings with the involvement of implementors and decision-makers while leading organisations could play a facilitation role in developing capabilities locally to accelerate integrations [40].

### Opportunity

*Opportunity* of the COM-B model was defined as physical and sociocultural factors creating conditions for action. The key barrier in this category was the differences in views between decision-makers and implementers regarding funding and resources in the Environmental context and resources domain. The other barriers related to opportunity were the social stigma related to cervical screening and the lack of preventative healthcare practices. The facilitator that emerged after the pandemic was the enhanced awareness and knowledge of viruses, diagnostics, vaccines and immunity.

#### Minimising gaps in resource allocations and improving screening programs with Information systems

Most decision-makers we interviewed expressed optimism about funding and resources, whereas most implementers identified limited funding and equipment for treatments as a significant barrier in screening programs. This disparity could potentially be due to the absence of comprehensive information systems to effectively reflect the impact and reach of screening programs. Only three out of the nine countries we interviewed had a screening registry. During the pandemic, many countries set up surveillance systems for outbreak monitoring. The expertise from deploying these information systems could be adapted to monitor cervical screening and vaccination programs. Bangladesh leveraged their information system to enable stakeholders to continue targeted outreach programs in areas with fewer COVID-19 cases and ensured continuity in screening programs [29]. Literature also underscored the importance of population-based cervical screening registries to map underserved communities and monitor the progress and quality of implementation [26, 41, 42]. The advantages of health information systems include making data-driven decisions through timely information on health determinants, cancer risk factors, and program performance, which would be crucial in minimising gaps in resource allocation and improving the screening program in the long term. However, to ensure the successful integration of any interventions, as mentioned by a decision-maker with experience in implementing a population-based information system, the success of a screening registry depends on the government’s support and comprehensive training at all levels, from community to district and from medical officers to health workers, to use the system effectively. Similarly, the effectiveness of integrating HPV testing depends on how well the information system aligns with the country’s health strategic goals and the availability of external financial, technical, and operational resources [4]. Hence, fostering better communication and collaboration between decision-makers and implementers would be the first and crucial step before introducing new strategies and interventions. Decision-makers and implementers could also work together to revise existing national guidelines, addressing barriers related to the continuum of care, risk stratification for treatment, and the choice of treatment options, such as thermal ablation versus cryotherapy.

#### Overcoming social stigma with self-sampling tests

The barriers mentioned by stakeholders and literature [43–46] were social factors such as a lack of preventative healthcare practices in the country and the stigma associated with cervical screening tests. On a positive note, stakeholders mentioned that after the pandemic, there was an increased understanding of viruses, diagnostics, vaccines, and immunity, as well as improved public and government attitudes towards preventive health. However, the intimate nature of the screening tests, which necessitates exposure to healthcare professionals and the associated discomfort, was a barrier mentioned by all stakeholders. In light of the increased public acceptance of self-sampling for testing purposes, some stakeholders believe now would be the opportune time to transition to self-sampling HPV tests. Self-sampling could be an alternative to clinician-collected cervical screening tests with successful pilots with high patient acceptability [47, 48]. A recent meta-review showed that self-sampling improved screening uptake, particularly in LMICs, but mentioned there were few pilots (six studies) included for study [49]. As mentioned by stakeholders, the decision-makers were aware of the HPV tests and the evidence related to acceptability in their or other countries. However, they mentioned the key barrier was related to cost and resources to switch from existing methods to implement HPV testing as the primary test. The review agreed with stakeholders’ concerns and called for cost-effectiveness analyses and further implementation studies on resource consumption related to HPV tests to support stakeholders in pushing forward the agenda. Hence, more work could focus on providing stakeholders with evidence-based roadmaps to introduce HPV tests and have more implementations in varying resources and settings. Some of the initiatives that are currently being developed include SHE-CAN (Self-collected HPV Evaluation for the Prevention of Cervical CANcer), the SUCCESS project (Scale Up Cervical Cancer Elimination with Secondary Prevention Strategy), and Project ACCESS (Accelerating Cervical Cancer Elimination with Self-Sampling).

### Framework strengths and limitations

The TDF was valuable for analysing qualitative data from semi-structured interviews and understanding the complex and evolving context of efforts like cervical screening programs. For instance, the framework has been employed to understand the behaviours of primary care practitioners regarding cervical screening and HPV vaccination in Ireland [20]. A systematic review also utilised the TDF as an analytical framework to examine perceived barriers and facilitators to cervical screening attendance [50]. Combined with the COM-B model, these frameworks effectively consolidated barriers and facilitators into domains and categories, clarifying their interrelationships to guide the next steps. However, it was challenging to categorise some barriers and facilitators into certain domains. For example, passionate stakeholders could be categorised under two domains–*emotions* and *optimism*. Another example was the shift in the government’s position towards preventive health services, which could be due to intentions and a change in beliefs about consequences in response to the pandemic. The framework was also limited in explaining their relationships in the changes in the domains before and after the pandemic.

Another limitation was the study sample size. Although we had good representation from various organisations in different LMICs, the study included only fifteen stakeholders. However, the team agreed that the data were deemed adequate after the fifteenth interview, as no new themes had emerged from both the decision-makers and implementers groups since the twelfth interview. Consequently, recruitment was halted to ensure the recency of the findings.

## Conclusion

Our findings indicated that most barriers related to cervical screening programs were not direct results of COVID-19 measures and existed before the pandemic. The successes of emerging facilitators implemented during the COVID-19 pandemic demonstrated opportunities to address some of the persistent barriers such as using social media platforms to raise awareness on cervical cancer and online platforms to train more healthcare providers in screening. However, further work would be required to align these strategies and interventions with each country’s health system goals and resource settings. A summary of opportunities was listed in Table 8 for actions. Stakeholders play a pivotal role in turning these strategies into actions. Closer collaboration between policymakers, decision-makers, and implementers would be a vital first step towards achieving the WHO’s target of screening 70% of women using a high-performance test at least twice in a lifetime by age 35 and again by age 45 [5].
