# Correction: From barriers to opportunities from COVID-19 pandemic: Stakeholder perspectives on cervical cancer screening programs in LMICs of the Asia-Pacific region

**DOI:** 10.1371/journal.pgph.0005067

**Published:** 2025-08-13

**Authors:** Jieying Lee, Ida Ismail-Pratt, Dorothy A. Machalek, Suresh Kumarasamy, Suzanne M. Garland

The corresponding author’s email address is incorrect. Ida Ismail-Pratt’s email is: idaipratt@hotmail.co.uk.
